# Acute Hormonal Responses to Multi-Joint Resistance Exercises with Blood Flow Restriction

**DOI:** 10.3390/jfmk8010003

**Published:** 2022-12-22

**Authors:** José Vilaça-Alves, Patrício S. Magalhães, Claudio V. Rosa, Victor M. Reis, Nuno D. Garrido, Rita Payan-Carreira, Gabriel R. Neto, Pablo B. Costa

**Affiliations:** 1 Sport Sciences Department, University of Trás-os-Montes e Alto Douro, 5000-801 Vila Real, Portugal; 2 Research Centre in Sports Sciences, Health Sciences and Human Development, CIDESD, 5000-801 Vila Real, Portugal; 3 Research Group in Strength Training and Fitness Activities (GEETFAA), 5000-801 Vila Real, Portugal; 4 Department of Veterinary Medicine, University of Évora, 7004-516 Évora, Portugal; 5 Department of Physical Education, Associate Graduate Program in Physical Education UPE/UFPB, João Pessoa 58051-900, Paraíba, Brazil; 6 Coordination of Physical Education/Professional Master’s in Family Health, Nursing and Medical Schools, Nova Esperança (FAMENE/FACENE), João Pessoa 58067-698, Paraíba, Brazil; 7 Coordination of Physical Education, Center for Higher Education and Development (CESED-UNIFACISA/FCM/ESAC), Campina Grande 58408-326, Paraíba, Brazil; 8 Exercise Physiology Laboratory, Department of Kinesiology, California State University, Fullerton, CA 92831, USA

**Keywords:** vascular occlusion, kaatsu training, resistance training, testosterone, cortisol, growth hormone

## Abstract

The purpose of this study was to investigate the acute effects of multi-joint resistance exercises (MJRE) with blood flow restriction on hormonal responses. Ten men participated in the study and underwent two experimental protocols in random order: four sets (30, 15, 15, and 15 reps, respectively) of MJRE (half squat and horizontal chest press) were performed with 20% of 1RM and a rest time between sets of 30 s, combined with intermittent blood flow restriction (LI + BFR protocol); and four sets (8, 8, 8, 20 reps, respectively) of the same MJRE performed with 75% of 1RM load (HI protocol), with a 90 s rest between the first three sets and 30 s between the third to the fourth set. Blood samples were collected before (PRE), immediately after (POST), and 15 min after the performance of MJRE (POST15). A time effect was observed for growth hormone (GH) and insulin-like-growth-factor-1-binding-protein-3 (IGFPB-3), but no protocol effects or interactions between protocol and times were observed (*p* > 0.05). There was no effect of either protocol or time (*p* > 0.05) on total testosterone, free testosterone, or cortisol concentrations. However, significant (*p* < 0.05) increases were observed in the GH serum concentrations of 2072.73% and 2278.5%, HI, and LI + BFR protocols, respectively, from the PRE to POST15 test. In addition, there was an increase of 15.30% and 13.29% in the IGFPB-3 concentrations (*p* < 0.05) from PRE to POST0 times for HI and LI + BFR protocols, respectively. Furthermore, there was a decrease of −6.17% and −11.54%, *p* = 0.00, between the times POST0 to POST15 in the IGFPB-3 for the HI and LI + BFR protocols, respectively. It is concluded that multi-joint resistance exercises combined with intermittent blood flow restriction seemed to promote acute hormonal responses in a manner similar to traditional exercise with high loads. Future studies may investigate whether chronic use of LI + BFR with MJRE may promote muscle hypertrophy.

## 1. Introduction

Resistance exercises (RE) with a high load percentage (≥70% one repetition maximum; 1RM) are often recommended to increase muscle hypertrophy [1]. However, in recent years, various studies have evaluated the use of low load percentages (20–50% 1RM) until volitional fatigue, or a higher number of repetitions (e.g., one set of 30 repetitions followed by three sets of 15 repetitions) combined with blood flow restriction (BFR), demonstrating increases in hypertrophy to a similar or greater extent than methods including higher load percentages but no BFR [2,3,4,5,6].

The increases in hypertrophy from the inclusion of BFR have been attributed to several factors, such as increased metabolic stress levels resulting from the accumulation of metabolites derived from muscle contraction [7], increased recruitment of fast-twitch muscle fibers [8], increased concentration of serum hormones [5,9], muscle edema [10], and increased production of reactive oxygen species (ROS) [8,11]. However, it should be noted that some of these mechanisms, in particular the increased fast-twitch muscle fiber recruitment and ROS production, are principally associated with high levels of mechanical stress, as seen in high-intensity (HI) RE, and not a result of metabolic stress [12,13]. Despite the low level of mechanical tension, it is possible that effects resulting from metabolic stress associated with BFR may induce muscle growth. Nevertheless, this mechanism needs to be better understood.

Studies using the BFR method have reported increased levels of specific hormones, especially growth hormones (GH) and insulin-like growth factor-1 (IGF-1) in men [5,9,14,15,16] and women [17,18]. Madarame et al. [16] demonstrated an increase of GH and IGF-1 post lower and upper limb exercises with BFR, with higher values of GH in the lower limb exercises. Takarada et al. [19] observed greater increases in GH concentration after BFR resistance training compared with a light load exercise protocol without BFR. Kim et al. [18] and Chen et al. [17] reported similar increases in GH between acute BFR resistance exercise and a HI protocol in young and postmenopausal women, respectively. In contrast, Manini et al. [15] demonstrated higher increases in GH after BFR resistance training compared with the HI protocol.

Abe et al. [14] reported increased IGF-1 following two weeks of training, including BFR with 20% of 1RM and greater increases in muscle hypertrophy compared with the group that did not receive BFR. However, the changes in muscle hypertrophy appeared to not have been associated with an increase in GH, free testosterone (FT), or IGF-1 in resistance training with BFR [20] and HI resistance training [21]. Similarly, Yasuda et al. [22] demonstrated an increase in muscle thickness of the triceps and pectoralis major with the performance of chest press exercises with BFR but with no significant increase in anabolic hormones.

Although the hypertrophy reported with resistance training with and without BFR is not associated with the serum increase in GH, testosterone, or IGF-1, the elevation of these hormones promoted an anabolic environment favorable to hypertrophy, regardless of the possibility of its occurrence. Thus, it is important to observe the effects of these training protocols on the acute anabolic and catabolic hormonal response.

Localized partial hypoxia and restriction on the venous return are the main mechanisms responsible for acute hormonal response to the BFR technique [23,24]. In the BFR technique, the cuffs are located in the axillary zone of the upper limb and the inguinal zone of the lower limbs, causing not all agonist muscles of multi-joint (MJ) exercises to be subjected to the effects of partial hypoxia and venous return restriction. It is taken into consideration that when analyzing the methodology of the acute effect studies, just the pilot study of Yasuda et al. [22] used only multi-joint exercises. For example, using the BFR technique in the bench press does not promote a decrease in blood flow or venous return to the major muscles involved in these exercises. Decreases in blood flow and venous return only seem to occur in the arm muscles that are smaller and with less intervention in the bench press. This fact can lead to lower metabolic stress induced by the BFR when compared with the same technique in the isolated exercises for the upper limbs and, consequently, a possible lower hormonal stimulation. Furthermore, exercises such as bench presses and squats are the most used in fitness facilities. Therefore, the findings of the present study would present a great application for health and sports professionals.

The relationship between hormonal responses and muscle hypertrophy remains a debated topic in the literature [20] and it has been suggested that HI and low-intensity (LI) plus BFR (LI + BFR) exercise may be associated with similar levels of hormone secretion [8]. Therefore, it is important to investigate the acute hormonal response resulting from RE, with and without BFR using MJ exercises. Therefore, the purpose of the present study was to investigate the acute effects of multi-joint resistance exercise (MJRE) with BFR on hormonal responses, specifically on levels of total testosterone (TT), FT, cortisol, GH, and insulin-like growth factor binding protein 3 (IGFBP-3).

## 2. Materials and Methods

### 2.1. Sample

The study sample consisted of 10 normotensive men between 19 and 28 years old who had practiced resistance training for at least 6 months, with a minimum frequency of 3 times per week (see Table 1).

Participants in the study were instructed to not take any nutritional supplements or alcohol and were asked to sleep for a minimum of six hours the night before testing. Finally, the participants were instructed to maintain the same eating habits throughout the study period. After the risks and benefits of participating in the study had been explained, participants read and signed an informed consent form, prepared in accordance with the Helsinki Declaration. The study was approved by the ethics committee of the University of Trás-Os-Montes and Alto Douro (protocol number 0476/13).

### 2.2. Study Design

**Figure 1:**
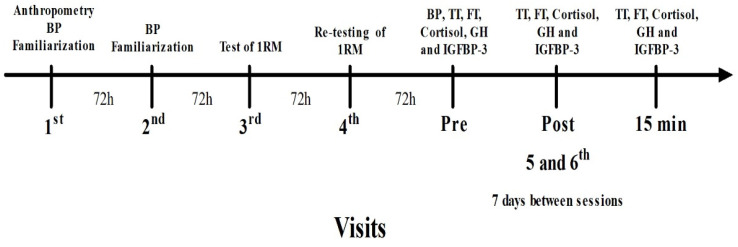
Study timeline. Legend: BP—blood pressure; TT—testosterone; FT—free testosterone; GH—growth hormone; IGFBP-3—insulin-like-growth-factor-1-binding-protein-3.

During their first visit, participants’ anthropometry and blood pressure were measured and they were familiarized with the exercises and BFR procedures. After 72 h, another familiarization session with the exercises and BFR was carried out. On the third and fourth visits (separated by 72 h), testing and re-testing of muscle strength were performed using the 1RM protocol for the half squat and bench press exercises. The first four visits were conducted between 8:00 and 11:00 am. Following these initial visits, participants visited the laboratory on two separate occasions seven days apart. The order of the two protocols was determined by a random crossover model. At each of these two visits, the participants arrived at the laboratory between 8:00 and 8:30 am, after 12 h of overnight fasting. Participants remained seated in a quiet room for 30 min prior to the collection of blood pressure (only for the LI + BFR protocol) and the first blood samples to measure hormone levels (PRE). Immediately after, participants were provided a standard 242 kcal snack consisting of 330 mL of water, 350 mL of orange juice, and a 60 g energy bar (Protein Chox Lemon Crunch Myprotein, Northwich, UK), which provided 22 g protein, 23 g carbohydrate, and 8 g fat. After 30 min, participants underwent the protocol, consisting of half squat and bench press exercises, with or without BFR, according to the treatment protocol. Blood samples were then collected immediately after the end of the session (POST0), and 15 min after (POST15), as shown in Figure 1. Hormones were selected due to their importance as markers of the body’s anabolic (TT, FT, GH, and IGFBP-3) and catabolic (cortisol) environments.

### 2.3. Blood Pressure Measurements

The blood pressure (BP) was measured using a mercury sphygmomanometer (Riester Ri-san^®®^, Jungingen, Germany) and a stethoscope (SpainCare KT-118, Valencia, Spain) by the auscultation method, always performed by the same experienced examiner. Measurements were performed according to the guidelines of the American Heart Association [25]. The SBPr was used for the occlusion during BFR training on the bench press exercise, and 120% of SBPr was used for the half squat exercise [26].

### 2.4. One-Repetition Test

The one-repetition maximum test was conducted for the half squat (90°) and bench press exercises with a conventional bar and calibrated weights. They were bilaterally conducted according the following techniques: (i) the participants performed a warm-up with a load that allowed five to ten repetitions; (ii) after a 1-min rest the participants performed three to five repetitions with a load increase of 5 to 10% or 10 to 20% of the load used in step one for the bench press and half squat, respectively; (iii) after a 2-min rest, a near-maximal load was estimated that allowed the participant to complete two or three repetitions by adding 5 a 10% or 10 to 20% of the load used in the step two for the bench press or half squat, respectively; (iv) after two to four minutes of rest, the participants performed a 1RM attempt by increasing the load used in step three by 5 to 10% or 10 to 20% for the bench press or half squat, respectively; (v) if the subject failed the 1RM attempt, a decrease of the load of 2.5 to 5% or 5 to 10% for the bench press or half squat, respectively, was provided until the participant could complete a repetition with appropriate technique; (vi) the participants 1RM was to be achieved within five attempts with two to four minutes of rest between them [27].

### 2.5. Blood Samples

Blood samples for hormonal analyses were taken before the exercises (PRE), immediately after (POST0), and 15 min post-exercise (POST15). At each collection, 10 mL of blood was obtained by an experienced nurse from an antecubital vein into serum gel tubes (S-Monovette^®®^; Sarstedt, Nümbrecht, Germany). Ten minutes after collection, the samples were centrifuged at 2000× *g* for 15 min, after which the serum was collected and stored at −20 °C. Serum concentrations of total testosterone and cortisol were determined using a chemiluminescent enzyme immunoassay system (IMMULITE^®®^ 1000; Siemens Medical Solutions Diagnostics, Los Angeles, CA, USA). The reactants were obtained from kits for cortisol and total testosterone (LKCO1 and LKTW1, respectively). Free testosterone, GH, and IGFBP-3 were measured by radioimmunoassay using a coated radioimmunoassay tube (KIPI19000; DIASource, ImmunoAssays SA, Ottignies-Louvain-la-Neuve, Belgium).

### 2.6. Experimental Sessions

Two REs consisting of half squats and bench presses (with a conventional bar and calibrated weights) were performed. Participants performed 2 protocols in a random order, one RE with 20% of 1RM combined with intermittent BFR (LI + BFR), and the other RE with 75% of 1RM (HI). Participants sat and rested for 30 min before both protocols. After this rest period, resting blood pressure was measured on the LI + BFR day and a blood sample was collected for hormonal assessment. Participants were then given a standard breakfast, and one of the protocols began 30 min later. For the LI + BFR protocol, participants completed 30 repetitions followed by 3 sets of 15 repetitions using 20% of 1RM, with a 30-s rest interval between each set. A standard blood pressure sphygmomanometer (Riester Ri-SAN^®®^; Jungingen, Germany) was attached to both legs (width 100 mm, length 540 mm) and arms (width 60 mm, length 470 mm) of each participant at the inguinal fold and the axillary fold regions, respectively. The cuffs were inflated for the duration of each set and were deflated between sets. Specifically, the cuffs were inflated after each of the 30 s rest intervals between sets until the completion of the last repetition of each set for a total duration of 105 s. The pressure used in the cuffs did not result in complete arterial occlusion but severely affects venous outflow, as reported by Patterson et al. [28]. For the HI protocol, participants completed 3 sets of 8 repetitions with 75% of 1RM, with a 90-s rest period between sets. Thirty seconds after the last set, one additional set of 20 repetitions with 40% of 1RM was performed. Participants were allowed to recover for up to 120 s between exercises. In both protocols, the execution cadence was set at 3 s (1.5 s for concentric muscle contraction and 1.5 s for eccentric contraction) which was controlled by a metronome (MA-30; Korg, Melville, New York, NY, USA).

### 2.7. Statical Analyses

The sample size was calculated based on procedures suggested by Beck [29] using G*Power software version 3.1 [30]. Based on a priori analysis, we adopted a power of 0.80, α = 0.05, correlation coefficient of 0.5, non-sphericity correction of 1, and an effect size of 0.50. From these values, an N of 10 subjects was calculated which corresponds to 80.3% of the statistical power.

A *t*-test for independent measures was used to examine possible differences between protocols (LI + BFR or HI) on training volume (TV), total protocol time (TPT), and time under tension (TUT). A two-way repeated measures ANOVA was used to examine the interaction between exercise protocols (LI + BFR or HI) and time points (PRE, POST0, and POST15). Possible significant differences between means were identified by the Bonferroni post hoc test. An analysis of covariance (ANCOVA) was used to determine the possible influence of covariates (TV, TPT, and TUT). Cronbach’s alpha intraclass correlation coefficient (ICC) was used to assess the reliability of the 1RM loads between test and retest. Effect sizes (ES) were calculated using partial eta squared (*η_p_^2^*) and Cohen’s d (*d* = difference between means ÷ pooled SD) for pairwise comparisons. Small, medium, and large ES would be reflected for *η_p_^2^* in values greater than 0.0099, 0.0588, and 0.1379, respectively, and for Cohen’s d in values greater than 0.2, 0.5, and 0.8 [31]. The level of significance was set at 5%.

## 3. Results

The ICC for tested 1RM of each exercise indicated excellent reliability with r = 0.98 and r = 0.97 for the half squat and bench press, respectively). Significant differences were found between protocols for TV (4731.00 ± 1271.76 kg versus 2542.50 ± 687.80 kg, *p* = 0.00, d = 2.14, HI and LI + BFR, respectively), TPT (788.70 ± 13.91 s versus 666.20 ± 4.21 s, *p* = 0.00, d = 11.92, HI and LI + BFR, respectively), and TUT (271.70 ± 10.59 s versus 456.20 ± 4.21 s, *p* = 0.00, d = 22.90, HI and LI + BFR, respectively). The HI protocol presented higher values of TV and TPT, and lower values of TUT than the LI + BFR protocol. The ANCOVA did not show significant (*p* > 0.05) interferences of TV, TPT, or TUT in the hormone concentrations between the different protocols and times.

No significant protocol—time interaction or protocol effect was found for any of the hormone concentrations (*p* > 0.05). However, there was a main effect for time for GH (Z_(2,18)_ = 9.044, *p* = 0.01, η_p_^2^ = 0.50) and IGFBP-3 (Z_(2,36)_ = 11.378, *p* = 0.00, η_p_^2^ = 0.56). Post hoc analyses demonstrated a significant (*p* = 0.03) increase in GH from PRE to POST15 of 2072.73% (d = 1.13) and 2278.5% (d = 1.42) for the HI and LI + BFR protocols, respectively. In addition, there was a significant (*p* = 0.01) increase in IGFPB-3 from PRE to POST0 of 15.30% (d = 0.45) and 13.29% (d = 0.43) for the HI and LI + BFR protocols, respectively. Furthermore, there was a significant (*p* = 0.00) decrease in IGFPB-3 from POST0 to POST15 of −6.17% (d = 0.19) and −11.54% (d = 0.41) for the HI and LI + BFR protocols (Table 2).

## 4. Discussion

The present study analyzed the acute effects of MJRE, with and without BFR, on hormonal responses. The primary finding of this study was that the LI + BFR and HI protocols induced similar hormonal responses. Both protocols increased GH, with significant differences between PRE and POST15, as well as IGFPB-3 between PRE and POST0. In addition, IGFPB-3 returned to baseline at POST15 for both protocols. All other hormones analyzed in this study showed no significant alterations.

The current findings concerning T, FT, and cortisol contrast with those reported by Gotshalk et al. [32], who reported a significant acute increase in cortisol and testosterone after a RE session with a high load percentage without BFR. This difference may be due to the lower training volume used in the present research compared with the aforementioned study. Moreover, the training volume seems directly related to increased concentrations of circulating anabolic hormones [32,33]. Alternatively, the findings of a study by Reeves et al. [34],^,^ which evaluated the effect of combined RE and BFR on TT, FT, and cortisol, were similar to those reported in the current study. These findings may also be related to the training volume, as this training protocol usually uses low load percentages (20–50% 1RM) with high repetitions, reducing the training volume compared with HI RE protocols. Another possibility is a fewer lower metabolic stress promoted by the bench press exercise in the BFR protocol. In the bench press exercise, the cuffs were on the axillar zone, and blood flow and venous return were only restricted only to the arms and not to the trunk. The principal’s primary agonist muscles of the bench press exercise are the chest, deltoid, and triceps brachii, and only the last exercise suffered the effect of the BFR. In addition, the mechanical stress induced on the triceps brachii is lower in the bench press exercise than in isolated elbow extension exercises. Hence, less accumulation of byproducts of glucose, micro-injuries, metabolic stress, and, consequently, less hormonal stimulation.

The results obtained for serum GH and IGFBP-3 were similar between protocols, which may have occurred because these responses are associated with both mechanical [32] and metabolic stress [24]. In addition, IGFBP-3 is the major IGF-I carrier protein, and IGF-I concentrations have been shown to increase with higher concentrations of blood GH [19]. The increase in IGFPB-3 for both protocols between PRE to POST0 and a return to baseline at POST15 are comparable to the results presented by Madarame et al. [16] and Chen et al. [17]. The increases in IGFBP-3 may be attributed to plasma volume reduction [35] after the exercises in both protocols (LI+ BFR and HI). Although there is a decrease in plasma volume, it occurs by different mechanisms. While with BFR, the decrease occurs due to the pressure of the cuff on the blood vessels, in the HI RE protocol, this decrease occurs due to the pressure exerted by the muscle fibers onto the blood vessels. The relief of the cuff pressure in the LI + BFR protocol and the decrease in muscle tension in the HI protocol in blood vessels leads to the occurrence of hyperemia, increasing plasma volume, which may be the reason for a decrease in the IGFBP-3 at POST15. In addition, it is necessary to consider that although the IGFPB-3 is the principal IGF-I binding protein, there are at least six binding proteins affecting IGF-I bioactivity, and the decrease in the IGFPB-3 serum levels may not necessarily represent a decrease in IGF-I bioactivity [36].

The GH increases at POST15 in the HI condition can be explained by the response of muscle fibers mechanoreceptors to mechanical stress [26]. In contrast, the combination of low-intensity RE and BFR increases the metabolic stress caused by the accumulation of hydrogen (H^+^) and inorganic phosphates resulting from anaerobic lactic metabolism and decreased venous return caused by the BFR [8,11,34,37,38]. The Accumulation of these metabolites resulting from muscle contraction, specifically in the sarcoplasmic reticulum, results in a “muscle pump” [37,38]. This causes activation of the cellular osmotic sensors and stimulates the hypothalamic–pituitary axis through group II and IV fibers (Madarame et al. 2010), providing an increase in serum GH concentration and, consequently, increased protein synthesis [24]. Indeed, the release of the tourniquet increases blood flow (hyperemia) and high secretion of metabolites from anaerobic lactic metabolism into the bloodstream, which are also potent stimulators of GH release [10].

Both protocols in this study appear to potentiate increased serum IGFBP-3. However, no significant differences were observed between protocols. This can be explained by the fact that both conditions promote mechanical and metabolic stress, while the principal mechanisms differ. The HI protocol promotes mechanical stress using relatively high loads, and the LI + BFR protocol uses high repetitions and, consequently, more time under tension. Concerning metabolic stress, it is achieved in the HI protocol by the increase in the difficulty of venous return and vascular restriction promoted by the higher tension produced in the muscular actions, and in the LI + BFR protocol by the higher time under tension, blood restriction, and increase in the difficulty of the venous return promoted by the cuffs’ pressure.

Both protocols could create a favorable anabolic environment conducive to increased protein synthesis and muscle hypertrophy. However, it should be noted that an acute increase in GH and IGFPB-3 does not necessarily induce muscle hypertrophy [20,39]. In addition, LI + BFR is associated with reduced joint and tendon stress, lowering the risk of osteoarticular lesions [8,11] and making it more suitable for specific populations than training with a higher load.

Finally, the present study had some limitations, one of which was the possible influence of the circadian rhythm of cortisol. The early morning peak and the subsequent decrease may have masked the actual effect of the exercise protocols [38,39] Another possible limitation was the blood pressure measurement performed while sitting, and the two REs were carried out in different positions. In addition, because the force of gravity promotes direct variation in hemodynamics when the individual changes his position [40], it becomes a limiting factor to evaluate the sitting BFR when proposing to perform exercises in other positions. However, the methodology methods used in the current study are similar to those observed in previous investigations [3,4,41,42,43,44]. Moreover, these results can only be extrapolated to subjects with at least six months of experience in resistance training.

## 5. Conclusions

In conclusion, the use of multi-joint high-load RE and low-load RE with BFR protocols resulted in similar acute hormonal responses. Thus, using MJRE combined with BFR by allied health professionals may be an alternative for individuals who cannot lift heavy loads but need to elevate their anabolic hormones. Therefore, it is important to conduct further studies to analyze different markers of acute and chronic hormonal pathways, especially those involving different BFR pressures, cuffs of different thicknesses, and varying load percentages in different populations.

## Figures and Tables

**Table 1:** Characteristics of the participants.

Variables	Mean ± Standard Deviation (*n* = 10)
Age (years)	22.50 ± 3.24
Height (cm)	177.30 ± 4.76
Body mass (kg)	72.20 ± 8.06
Estimated body fat (%)	8.23 ± 2.53
Systolic blood pressure (mmHg)	121.40 ± 4.55
Bench press (kg)—1RM	61.50 ± 18.27
Half Squat (kg)—1RM	108.00 ± 28.98

**Table 2:** Serum hormone concentrations (mean± standard deviation) in the two protocols before (PRE), immediately after (POST0), and 15 min post-exercise (POST15).

Variables	Protocols	PRE	POST0	POST15
TT (nmol.L^−1^)	HI	20.10 ± 13.92	15.30 ± 6.70	14.59 ± 5.29
	LI + BFR	16.50 ± 7.29	14.40 ± 5.32	16.74 ± 13.71
FT (nmol.L^−1^)	HI	11.75 ± 4.96	13.41 ± 3.95	11.17 ± 3.58
	LI + BFR	11.31 ± 3.83	11.95 ± 4.34	11.52 ± 3.85
Cortisol (nmol.L^−1^)	HI	359.50 ± 107.33	347.08 ± 141.51	354.81 ± 155.31
	LI + BFR	399.51 ± 135.82	461.00 ± 141.06	429.58 ± 151.29
GH (μg.L^−1^)	HI	0.11 ± 0.08	0.93 ± 1.28	2.39 ± 2.85 *
	LI + BFR	0.14 ± 0.13	2.05 ± 2.46	3.33 ± 3.118 *
IGFBP-3 (mg.L^−1^)	HI	4.64 ± 1.37	5.35 ± 1.79 !	5.02 ± 1.66 **
	LI + BFR	4.59 ± 1.28	5.20 ± 1.56 !	4.60 ± 1.33 **

TT—total testosterone; FT—free testosterone; GH—growth hormone; IGFBP-3—insulin-like growth factor binding protein 3; HI—high-intensity protocol; LI + BFR—low-intensity with blood flow restriction protocol; *—*p* = 0.03 in relation to the time PRE; **—*p* = 0.00 in relation to the time POST0; !—*p* = 0.01 in relation to the time PRE.

## Data Availability

Data available on request from the corresponding author.
